# ‘It stretches your body but makes you feel good too’: A qualitative study exploring young people’s perceptions and experiences of yoga

**DOI:** 10.1177/13591053221146840

**Published:** 2023-01-12

**Authors:** Tina Cartwright, Tatjana Doronda

**Affiliations:** University of Westminster, UK

**Keywords:** adolescents, experiences, qualitative, self-regulation, yoga, youth

## Abstract

Whilst research suggests that yoga can positively impact physical and psychological wellbeing, understanding of youth’s experiences is limited with no non-clinical studies in the UK. Ten focus groups explored perceptions and experiences of yoga among 35 youth (10–18 years). Inductive thematic analysis revealed that yoga was viewed as a holistic mind-body practice cultivating greater awareness and enhanced physical performance. Youth described yoga as providing tools that developed confidence, stress-management and emotional self-regulation. Social and relational impacts of yoga were highly valued. Despite the perceived biopsychosocial benefits of yoga, gendered and media representations of yoga may serve as a barrier to uptake.

## Introduction

Adolescence represents a sensitive period of biological and social development, and one which is critical for developing socio-emotional skills for future adult life. It is also a period of multiple stressors, including interpersonal and academic pressures, with chronic stress associated with increased risk of mental health and emotional difficulties (Roberts et al., 2009). A recent representative survey in the UK suggests that 13% of children and adolescents have a diagnosed mental health condition (Sadler et al., 2018), yet access to support such as Child and Adolescent Mental Health Services is limited. Mental health conditions frequently persist into adulthood (Kessler and Wang, 2008), and are associated with other negative outcomes such as poorer academic achievement, self-harm and health risk behaviours (Sadler et al., 2018). Given these challenges and the importance of supporting adolescent wellbeing, there has been increasing focus on developing youth programmes that promote mental wellbeing and develop resilience and life skills to manage stressors (Mackenzie and Williams, 2018; Sawyer et al., 2012).

Mind-body programmes, such as yoga and mindfulness, focus on the ‘interactions among the brain, mind, body, and behavior’ and the ways in which they can directly affect health (Morone and Greco, 2007: 360). They are gaining in popularity to promote wellbeing, with growing research demonstrating a range of positive outcomes for young people (Miller et al., 2020). Yoga can be viewed as a complex health intervention, combining physical postures, breathing exercises, relaxation techniques, meditation/mindfulness practices and practical yoga philosophy. These practices, both individually and in combination, have been found to improve mind-body awareness, physical and respiratory functioning, attention and self-regulation skills in both adults and young people (Gard et al., 2014; Khalsa and Butzer, 2016; Schmalzl et al., 2020). Physiologically, it is suggested that yoga reduces stress through down-regulation of the hypothalamic-pituitary-adrenal axis and sympathetic nervous system (Pascoe and Bauer, 2015; Pascoe et al., 2017). Psychological mechanisms include learning more adaptive responses to stressors, and increases in mindfulness and self-compassion (Riley and Park, 2015). Socially, yoga offers an opportunity to connect with others and develop a sense of belongingness and acceptance (Cheshire et al., 2022; Kinser et al., 2013). Regular yoga practice may thus offer unique benefits for youth through increasing resilience and supporting wellbeing (Khalsa and Butzer, 2016; Mendelson et al., 2014).

There have been several systematic reviews of the effectiveness of yoga interventions for young people’s physical and mental health, predominately focusing on school-based interventions. Two reviews on yoga in school settings identified a range of physical and psycho-emotional benefits in studies using both self-reported and objective outcome measures. They also highlight the growth of interest in this field with Khalsa and Butzer’s (2016) bibliometric analysis identifying 47 publications compared with Serwacki and Cook-Cottone’s (2012) 12 studies. A recent systematic review focusing solely on RCT designs, included 39 studies across a number of settings and seven countries, with 26 in schools and the majority either in the US (*n* = 18) or India (*n* = 16), with none in the UK (Miller et al., 2020). Miller et al. categorised the broad range of outcomes into three domains: psychological/behavioural, cognitive and physiological/physical functioning, with 34 studies demonstrating improved outcomes in at least one domain. Positive effects on psychological outcomes were most commonly reported, including improvements in self-esteem, self-regulation, resilience and coping, with decreases in negative affect, mood disturbance and anxiety. Cognitive effects included improved memory and executive functioning, whilst a number of studies explored a range of physiological changes following yoga, such as fitness, balance and respiratory functioning.

Looking more specifically at the potential of yoga for reducing symptoms of anxiety and depression in youth, a recent systematic review found that 70% of the 27 studies demonstrated improvements in either/both depression and anxiety symptoms (James-Palmer et al., 2020). Whilst such research is promising, the above reviews highlight the weak to moderate methodological quality of studies in this relatively new field. For example, small samples, lack of randomisation, lack of active controls and inadequate reporting of yoga interventions. Qualitative research is particularly valuable for providing a more nuanced perspective on the impact of yoga, whilst enabling insight into how young people make sense of and attribute value to their experiences (Wang and Hagins, 2016). Such understanding is also important to unravel potential mechanisms by which yoga may impact on youth’s health and wellbeing, for example through the implementation of skills in everyday life (Dariotis et al., 2017).

Several existing qualitative studies have explored the acceptability and impact of school-based yoga programmes. These have all been nested in controlled trials in the US, with yoga programmes ranging from 8 to 32 sessions and including youth between the ages of 11 and 16 years (Butzer et al., 2017; Case-Smith et al., 2010; Conboy et al., 2013; Dariotis et al., 2016; Wang and Hagins, 2016). Consistent with quantitative findings, emotional regulation and stress management were the most widely discussed benefits of yoga. Importantly, across all studies youth re regulation and stress management were ported reductions in emotional reactivity and impulse control (e.g. decreased anger and negative emotions) suggesting the application of regulation skills to everyday life. Indeed, Dariotis et al. (2016) specifically asked their participants to reflect on the skills learnt and implemented after the programme, with youth describing a range of strategies, breathwork in particular, to ‘de-escalate negative emotions, promote calm, and reduce stress’ (p. 76). For some, this skill set was perceived to positively impact on academic performance, particularly through the ability to concentrate and focus alongside manage the stress of exams (Conboy et al., 2013; Wang and Hagins, 2016). In other studies, participants described improvements in mood and optimism (Conboy et al., 2013), self-esteem (Case-Smith et al., 2010; Wang and Hagins, 2016), confidence (Wang and Hagins, 2016) and sleep (Butzer et al., 2017; Conboy et al., 2013). On a physical level, improvements in athletic performance were noted in several studies (Butzer et al., 2017; Conboy et al., 2013; Wang and Hagins, 2016), most commonly in terms of flexibility, strength and balance, but additionally in body awareness (Case-Smith et al., 2010; Conboy et al., 2013).

Novel to qualitative studies was the finding that yoga impacted on social relations for some youth (Butzer et al., 2015; Conboy et al., 2013; Dariotis et al., 2017), including improved relationships and an enhanced awareness of others needs and emotional states which promoted more positive social interactions (Dariotis et al., 2016, 2017). Some youth discussed how shared experiences within the yoga class promoted peer bonding, whilst others found such intimacy intimidating, particularly in mixed gender classes. Despite positive benefits reported across all studies, limitations included adequate time to integrate skills (Butzer et al., 2017), preference for other physical activity (many of the interventions were implemented in physical education classes), and gender issues around acceptability of yoga, for example, males in one study reported ‘peer pressure to not like yoga’ (Conboy et al., 2013: 176).

Taken together, the majority of research on yoga in youth has been conducted in the US and India, with none of the reviewed studies conducted in the UK, which is surprising given calls to develop programmes to promote wellbeing and reduce the risks of mental ill-health in young people in the UK (Mackenzie and Williams, 2018; Sawyer et al., 2012). Whilst previous qualitative research has provided insights into the acceptability and impact of yoga interventions in US schools, we currently know very little about young people’s attitudes towards yoga and their experiences of practicing yoga in community settings and in a UK context. In this qualitative study, we were interested in the perceptions and experiences of young people choosing to practice yoga in the UK. Specifically, the present study aimed to explore young people’s understanding and attitudes towards yoga, their experiences of practising yoga and any perceived impact on their health and wellbeing.

## Method

### Recruitment

An advertisement about the study was placed on several Facebook groups providing yoga to youth: Teen Yoga Foundation, Yoga Teachers UK and Teen Yoga Teachers’ Forum. Inclusion criteria were: 10–18 years old, with a minimum of 2 months (at least weekly) yoga practice; with no diagnosed mental health condition. Whilst a large number of yoga teachers and several parents responded to the advertisement, many of these programmes had stopped over the summer period at the time of recruitment (June–July 2019). Three yoga teachers, two from London and one from North England, and three parents agreed to facilitate recruitment.

Yoga teachers advertised the study at their yoga studios. Information about the study and consent forms were given to the parents of youth interested in participating in the study. Young people were given a participant information sheet and consent form, with time to discuss their choice to participate with a trusted adult. Both parents and participants provided informed consent, were fully informed about the purpose of the study, how their data would be used and how they could withdraw their consent. The study was approved by University of Westminster Psychology Ethics Committee.

### Participants’ characteristics

Thirty-five young people (27 females, 7 males) attending school or community-based yoga programmes agreed to participate. Participants were aged between 10 and 18 years (mean = 13 years), the majority (33) were White British, with one Black British and one British Indian. Most had practiced yoga for less than 1 year (*N* = 22), 11 between 1 and 2 years and 2 more than 2 years, with 77% (*n* = 27) practising yoga once a week, 23% (*n* = 8) practising more than once a week (see Supplemental Material for further sample details). Three males were not currently practising yoga.

### Data collection

Focus groups were selected over individual interviews since previous research suggests they are more acceptable to youth, help to reduce power differentials between researcher and youth and encourage open discussion with peers (Kirk, 2007; Punch, 2002; Wilkinson, 1998). Ten focus groups were conducted by TD in London and the south-east, Manchester region and West Yorkshire. Three took place at the homes of students (in arrangement with parents who had responded to the study advert), with the remainder conducted at the yoga studio where participants practised yoga. Focus groups included 2–5 participants, with one exception where there were nine participants (where all participants in a class wished to take part). Neither parents or yoga teachers were present during focus groups and no participants withdrew from the study.

The focus group topic guide included questions relating to perceptions of yoga (e.g. what does yoga mean to you personally?), experiences and effects of yoga (e.g. how does yoga make you feel?), attitudes and use of different components of yoga (e.g. what aspects of a yoga class do you enjoy most, why?), and barriers to yoga practice (‘what are the barriers to practicing yoga?’) (see Supplemental Material). Before each focus group, participants were reminded about the purpose of the study and their right to withdraw. Demographic and yoga practice data was recorded and each participant was asked to choose a pseudonym. Each focus group was audio-recorded and lasted around 30 minutes.

### Data analysis

Thematic analysis, a method for ‘identifying, analyzing and reporting patterns within data’ (Braun and Clarke, 2006: 79), was used to analyse the data, informed by a critical realist approach. Critical realism assumes that data provides access to participants’ ‘reality’ but that our interpretation of this reality is inevitably imperfect (Willig, 2012). Thus, our study sought to understand youth’s perspectives and experiences of yoga whilst acknowledging that the analytic process is inevitably shaped by the lens of the researcher. Data was analysed inductively and was data led, giving focus to the voices of young people, rather than any pre-conceived ideas about yoga and its effects. In line with Braun and Clarke’s (2006) six-phase guide, each transcript was read, re-read and then independently coded manually line by line by the researcher (TD). The codes representing recurring ideas, were subsequently categorised into preliminary themes and sub-themes. At this stage, the first author (TC) read the transcripts and reviewed coded extracts relating to each preliminary theme, leading to further development of subthemes and hierarchical structuring of the themes. Both authors then reviewed themes for their coherence and overall relevance to the research question and agreed the final thematic structure.

The interviewer (TD) was a yoga teacher trainee during the research. The researcher’s personal interest in yoga was not disclosed to participants. To ensure rigour, original transcripts were repeatedly returned to, in order to check preliminary interpretations and codes against original accounts. Analyst triangulation was also employed at initial and later stages of analysis. Qualitative reporting guidelines were followed to enhance the rigour and transparency of the research (Tong et al., 2007).

## Findings

**Figure 1.**
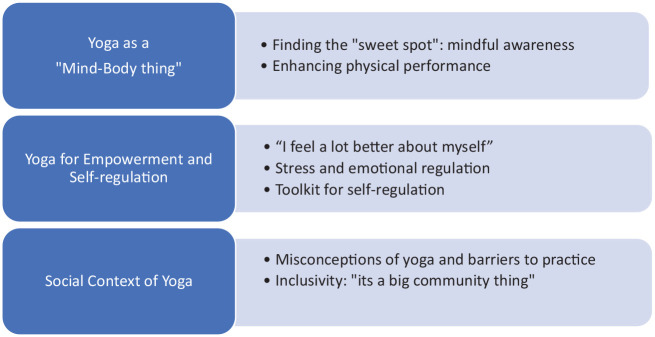
Summary of themes and subthemes.

Three main themes were identified (see Figure 1) which revealed: (1) youths’ perceptions of yoga as a mind-body discipline cultivating greater awareness and enhancing physical performance; (2) a practice offering tools that empowered youth to self-regulate stress and difficult emotions; (3) the social barriers and facilitators to uptake and maintenance of yoga practice.

## Theme 1. Yoga as a ‘mind-body thing’

Across all focus groups, youth defined yoga as a method that engaged mind and body, both in terms of techniques and impact. Whilst some gave greater emphasis to physical (strength, flexibility, balance) or mental effects (‘free your mind’), the majority saw the two as inexorably linked: ‘it stretches your body but makes you feel good too’ (Alice, 13, FG8).

I think yoga is like. . . physically calming your body, but also mentally. Like. . . when you feel physically calm, you are mentally calm. (Kate, 15, FG8)

There were interesting discussions around defining yoga, with many using sport as a comparator whilst highlighting differences, primarily in terms of mind-body benefits – ‘I think it is on the boundary between sport and something completely different’ (Vector, 14, FG1).

It’s a sport but it’s helping my flexibility as well. It is also helping my mind, but just differently to other sports, I think. In a way that I can just clear my head as well. (Becky, 13, FG4)

Yoga was perceived to develop skills and abilities beyond physical enhancement. For example, physically challenging balancing poses, such as the ‘crow’, were described as increasing body awareness, necessitating mental and physical focus. Interestingly, yoga was described as a ‘gateway for getting into other things like the meditating. . . yoga is kind of a bridge between the two things’ (James, 15, FG3).

### Finding the ‘sweet spot’: Mindful awareness

I feel that ever since I’ve started I’ve really seen a difference in my moods and where my mind is. (Eloise, 13, FG1)

Yoga was described as a practice for getting into ‘your own zone’ and escaping into ‘another world where you can be calm and relaxed and be yourself’ (Tia, 10, FG7). Participants described yoga as providing a mental space where they could see and understand themselves better, acknowledge their worries and ‘clear the head’. In contrast to exercise, yoga was seen as ‘for yourself’, with participants reporting feeling ‘levelled and centred’, ‘balanced’, ‘happier’ and ‘free’ following yoga.

All participants associated a feeling of ‘calm’ with yoga – this was distinguished from ‘sleepy relaxed’ and instead referred to as ‘more awake and like, aware. . .’ (Sophie, 13, FG6). Participants articulated how yoga gets them ‘to almost that neutral state of body and mind’ and described this feeling as being ‘between tired and alert’ which ‘sort of centers you’ (Alan, 15, FG3). When questioned further about this state, participants described how various mindfulness-based practices in yoga taught them to be ‘rooted’ in the present moment and to embrace their surroundings ‘from all the senses’. When asked why it is important to be ‘clear and mindful’ and how mindfulness helped in their day-to-day life, some participants suggested it helped them be more focused and able to concentrate. Others suggested it provided perspective: ‘Yoga makes you think about most important things, not focus on the least important’ (Quentin, 15, FG6).

### Enhancing physical performance

Many participants reported how yoga complements and enhances their physical performance in other athletic activities, like dancing, football, running and swimming. Yoga was additionally described as ‘something you can do for your body to make sure you are healthy’ (Lola, 12, FG6). Most mentioned how yoga postures promoted greater flexibility (‘more fluid almost’) and prevented tight muscles and injuries. Several girls who were dancers suggested that breathing helped with calming down before performing, while yoga postures were used for warming-up and stretching, all of which enhanced their physical performance. Sophie highlighted the importance of proprioception (sense of body position and movement in space) for movement in yoga compared with other physical activity:

Yeah, normally if you stretch you are told: “oh, if you are stretching things out if it hurts it means it’s working”. But it’s kind of different at yoga, they always tell us you shouldn’t’ be hurting, it should just be to the point when you feel like, your body is in the right place. (13, FG6).

Breathing techniques learnt during yoga were extended to enhance performance in other activities as well as to balance energy levels - ‘if I’m out for a run if I keep my inbreath longer and that’s giving me more energy’ (Tim, 18, FG5). All participants made a clear distinction between sports and yoga, contrasting the non-competitive and calming nature of yoga (‘a calm kind of way of fitness’), with other exercise, which ‘kind of stress you out’ (Sophie, 13, FG6).

## Theme 2. Yoga for empowerment and self-regulation

The majority of participants discussed how yoga had given them tools to manage the day-to-day stressors of school and cope specifically with the emotional demands of being a young person. Participants discussed ways in which yoga helped reframe difficult situations, increased their confidence and empowered them to take an active role in their mental health.

I think, to me, like, personally yoga has really helped. I go to therapy for situations and I think yoga for me has been more empowering like, I feel like, I’ve been in more control of what my mind is thinking. . ..it’s very important for young people to feel like, no matter the situation, like they are in control of what they can do. (Kate, 15, FG8)

### ‘I feel a lot better about myself’

The ‘mental space’ described in the first theme appeared to facilitate greater self-awareness which helped participants to feel more at ease and kinder towards themselves:

It makes you feel like. . . For me personally, I feel more open. . .more comfortable with myself, so like. . . I feel a lot better about myself. (Maxeine, 14, FG2)

Many participants highlighted the sense of accomplishment arising from progress in their yoga practice. Participants compared what they could do now, in terms of yoga poses, to when they started, which helped in building self-confidence. In some cases, participants discussed how yoga had increased their awareness and appreciation of their body which felt empowering. Contemplative practices, like meditation and relaxation, were perceived as particularly supportive of self-acceptance.

You kind of, instead of thinking - oh, I need to push myself harder! It’s more like - oh, I just need to be thankful for what I’ve done! This kind of makes you feel proud almost because you’ve done something for yourself. (Sophie, 13, FG6)

Additionally, many participants reflected how they felt more confident when faced with difficult situations, employing yoga techniques to put ‘everything into perspective’. This could have a powerful impact on their self-perceptions and the extent to which they were able to release self-judgement.

Before I went to yoga class, I always felt quite depressed. . . When I started coming and bad things happening outside of the yoga class, I always remembered to do some mindful breathing and it always helped me a lot. (Tia, 10, FG7)

### Stress and emotional regulation

All participants described school commitments as a major stressor in their lives, especially during exams. Practicing yoga during this time was perceived as ‘massively stress relieving’ (Tim, 18, FG5). Additionally, many reflected more broadly how young people feel ill-equipped to deal with stressors associated with being a teenager, ‘when the most stress starts’ (Mary, 12, FG4).

Yeah, I think it’s [yoga] more important for young people because. . . definitely for me, I’m stressed out about the future. . . And for younger people it’s quite hard to emm. . .I think, get over stress. . .they don’t maybe know how to cope with certain things. (Olivia, 15, FG8)

Most commonly, yoga was described as providing the opportunity for ‘letting go’ of worries and concerns and regaining equilibrium. Participants appreciated how yoga helped them to take a more active role in dealing with day-to day stressors, giving examples of how this enabled them to make better decisions and deal more effectively with challenging situations.

Our yoga teacher says if we are feeling stressed and we just want to like calm down we can go somewhere quiet and you can just practice that breathing. . . I think as well when you are stressed you can do stuff you probably don’t mean. So, then you can just calm down a little bit and then you will be more yourself, calm. (Becky, 13, FG4)

Some described how taking a yoga class provided a ‘escape’ from thinking about problems (e.g. homework), enabling a shift from ruminating to doing – ‘I’ll just have some peace. . . and then I’ll go home and do my homework’ (Olivia, 15, FG10). Others discussed how yoga had helped them to reframe difficulties by relaxing and allowing time to ‘think things through’, reflecting they felt able to ‘deal with situations a little better, because I have that sort of way of calming myself down’ (Maxeine, 14, FG2).

Others explicitly discussed how yoga helped them to dealing with difficult emotions, such as feelings of anxiety or low mood, helping them to manage their mental health and prevent the establishment of more permanent mental health issues - ‘yoga like, detoxes you’ (Lea, 15, FG10). More broadly, participants discussed development of skills of self-awareness and self-soothing to support emotional self-regulation:

Before I came to yoga, I used to get quite frustrated a lot, like at home and after school when something happened. . . . But when I came to yoga, I started to feel better about myself and it made me a little bit more confident. So, now I don’t get as upset and if I do, I just do some breathing and do some poses. . . (John, 10, FG7)

Whilst yoga provided an opportunity to let go and feel calmer in the midst of external demands, some participants acknowledged that these effects were not necessarily long-term:

It’s not like, a long-term effect that I feel. . . But I do feel like, relaxed and I don’t worry about homework or friendship. (Sam, 13, FG9)

### ‘Toolkit’ for self-regulation

Participants talked about using yoga techniques as a ‘toolkit’ for self-regulation and described a range of different methods, as touched on in previous themes. During the focus groups, participants frequently demonstrated popular poses and breathing techniques, describing their functions and how they were applied. For example, poses for calming and improving sleep (e.g. Child’s pose), and one mentioned using Warrior Pose for building her confidence. Some students recalled different types of meditation they used outside of their yoga class.

Yeah, I feel like breathing is one of the yoga’s main thing, and meditation. You can use them at school or at work, go to the toilets, and just sort of breathe or meditate and you know, even just do it at class, wherever you are. (Eloise, 13, FG1)

Breathing techniques were discussed by virtually all participants, described as particularly useful for calming and concentrating during exams, when feeling emotionally overwhelmed, angry, anxious or depressed. Young people talked about the effectiveness, simplicity and accessibility of those techniques in their daily life.

Well, before yoga when I was feeling anxious, I didn’t really know how to deal with it, but after it I learnt how to breathe. (Mary, 12, FG4)

A range of breathing techniques were described, including ‘Ujjayi’ breath (‘we call it Darth Vader breath’), ‘breathing through your tummy’, controlling the length of the inbreath and outbreath (‘the 4,4,4’) and mindful breathing. Interestingly, even male participants who no longer attended classes discussed how they had retained the breathing techniques which helped ‘control myself better in the long-term’ (Alan, 15, FG3).

## Theme 3. The social context of yoga

This theme explored initial barriers to yoga uptake amongst young people, reflecting the influence of media portrayal of yoga and youth’s concerns about fitting in. This was contrasted with actual experiences of yoga which was perceived as inclusive and supportive of building social relationships which facilitated continuation of practice.

### Misconceptions about yoga and barriers to practice

Initial perceptions of yoga were strongly influenced by media representations of yoga such as online pictures of challenging poses, as well as negative peer attitudes (‘they said yoga, that’s for old people’, Tillyboo, 10). Perceptions of ‘not being physically or mentally flexible’ for yoga, served as an initial barrier to yoga practice for many participants. Prior to beginning yoga, many accounts reflected the negative impact of social comparisons, including fear of being judged by others, not being ‘good enough’ and feeling self-conscious practicing with others who were perhaps more physically able.

‘I was really scared of being judged when I went, cause I’m not like the most flexible person, I can’t do everything’. (Alice, 13, FG8)

These fears were still evident in participants initial experiences but were short-lived; participants described how regular attendance and increasing self-confidence removed their social anxieties and changed their perceptions of yoga.

So, I think anybody can do yoga, even if you don’t think you gonna be very good at it. . .you learn. . . cause your teacher is gonna teach you. (Bobby, 10, FG7)

Reflecting on their own initial misperceptions about yoga, young people spoke about the importance of raising ‘awareness of what yoga classes actually are’ in order to engage youth and counteract public perceptions of yoga (‘more than the flexibility of your body’). More specifically, participants felt it was important to highlight other components of yoga, such as breathing and relaxation, along with the broader benefits for mental and physical health.

Interestingly, peers’ perceptions of yoga as ‘kind of weird’, ‘easy’ or ‘boring’, did not serve as barriers, instead participants were keen to share how yoga might help their friends in other activities such as dancing. Whilst male participants acknowledged that some of their school friends might not consider yoga ‘cool’, they were initially more concerned about ‘this fear that you’ll be like gender-swamped (dominated by females)’ (James, 15, FG3) and not being able to make friends. Attending with friends and subsequently making new friends was an important motivator for boys in this study, as highlighted by Vector who had given up yoga when his friends stopped attending classes:

My friends did go there, but then slowly they dropped out. I was the only guy. Maybe if they hadn’t dropped out, I would be still doing it today. (14, FG1)

### Inclusivity: ‘It’s a big community thing!’

Participants discussed extensively the friendship groups created from yoga, and the value placed on helping and supporting each other during the practice. Yoga was seen as more pro-social and inclusive compared to other sports, which brought ‘out a lot of good in people’ (Lea, 15, FG10). Participants discussed how they ‘share yoga’ in classes, by assisting and helping each other in stretches, doing partner poses and working in groups (‘it’s more cooperative than competitive!’). Several participants spoke about sharing yoga with their friends and family outside of the yoga class, expressing enjoyment at seeing others gain benefit from yoga.

Most participants viewed the yoga teacher as an important figure who ‘can sort of make or break the practice’ (Alan, 15, FG3). Several discussed the value of the teacher’s ability to be responsive to the specific needs of the students (‘she will base the yoga on how we feel’, Lea, 15, FG10), supported by smaller class sizes. Younger participants also appreciated teachers’ who were able to make the class fun, allowing students to ‘let go’. Here Sam describes his experiences with a previous teacher:

. . .she would make us do lots of silent meditation, we didn’t really enjoy that because we just couldn’t let go and it would just make me like stressed. (13, FG9)

Teacher-student trust was described as important for enjoyment and in facilitating greater openness in youth (‘makes it more real’). Participants also spoke about building trust with other students, particularly during group work, which helped them to develop their own practice (‘they can help you get higher’) as well as build their social skills:

Yeah, it can help you with like, with your social life, cos you have to talk to other person and trust that they know what they are doing. (Gertrude, 11, FG6)

## Discussion

To our knowledge, this is the first study that has explored perceptions and experiences of yoga among young people practising in school/community settings in the UK. Youth of all ages described yoga as a holistic mind-body practice which cultivated greater awareness and mental clarity as well as enhancing physical performance. Techniques learned during yoga, particularly breath regulation and meditation, were perceived as empowering tools for boosting self-confidence, stress-management and emotional self-regulation. Participants described how they were able to translate these skills into everyday life situations to help them better manage the challenges of adolescence and school pressures. A key finding was the social and relational impact of yoga participation. The inclusive and supportive nature of yoga was highly valued for building social skills, developing friendships and ensuring enjoyment of classes. However, gendered and media representations of yoga shaped initial perceptions, raising concerns about being ‘gender swamped’ for boys and lacking the physical flexibility and capability to practice yoga. The study thus highlights the need, as highlighted by youth themselves, to raise awareness of the biopsychosocial impact and inclusivity of yoga to counteract media stereotypes and reduce potential barriers to practice.

These findings are broadly consistent with previous qualitative studies investigating experiences of yoga programmes in school settings in the US (Conboy et al., 2013; Dariotis et al., 2016; Wang and Hagins, 2016). However, few previous studies have explicitly explored youth perceptions of yoga; we found that participants tended towards a holistic view of yoga, recognising the inter-relationship between mental and physical components and impacts of yoga, which was evident even amongst younger participants. This contrasts with Conboy et al.’s (2013) evaluation of a 12 weeks yoga intervention, where participants predominately focused on either physical or mental benefits. This may reflect a more sophisticated understanding arising from greater engagement with yoga based on practice duration and commitment given that our participants had chosen to practice yoga as opposed to undertaking a school-based intervention. This adds to previous calls to systematically evaluate yoga dosage requirements for maximal and long-term benefit, since some participants in our study noted that positive impacts were relatively short-lived (Dariotis et al., 2016; Miller et al., 2020).

As in previous studies, our participants described feeling ‘calmer’, but also reflected in detail how yoga helps them to establish mental equilibrium with greater attention to the present moment. Mindfulness is widely defined as consciously bringing attention to present moment experiences with an attitude of curiosity and acceptance (Bishop et al., 2004). Young people describe learning mindfulness skills through both contemplative and postural practices, for example staying with sensations in more challenging poses. It is suggested that engagement with body sensations develops focused attention and response inhibition, thus enabling a way of reframing challenging experiences (Gard et al., 2014). Participants in our study noted greater clarity, focus and concentration as well as perspective-taking. Importantly, young people discussed how greater self-awareness led to reductions in self-judgement and increases in self-acceptance, positively impacting on mood. This is consistent with the broader literature demonstrating positive associations between mindfulness, self-compassion and psychological wellbeing (Biber and Ellis, 2019; Keng et al., 2011).

In line with previous qualitative (Case-Smith et al., 2010; Dariotis et al., 2016; Wang and Hagins, 2016) and quantitative studies (Miller et al., 2020), yoga was widely perceived to benefit physical health and enhance performance in sporting activities, with increases in flexibility and breathing capacity. Whilst these benefits were largely seen as by-products of the yoga practice, this could serve as a useful ‘pull factor’ to engage young people, especially males, given consistent findings that both acceptability and uptake of yoga is lower in males (Conboy et al., 2013; Webb et al., 2020). Emphasising the impact of yoga on athletic performance, strength and flexibility may thus provide a ‘gateway’ to broader socio-emotional and self-regulatory benefits (Wang and Hagins, 2016).

The development of self-regulation skills is proposed as a key mechanism by which yoga leads to positive health outcomes in young people (Butzer et al., 2016) as well as adults (Schmalzl et al., 2020). Young people in the current study gave specific examples of how they felt better able to recognise and deal with difficult emotions (such as anger, anxiety and low mood), reframe problems and make better decisions. Importantly participants perceived yoga as providing them with highly valued agency by offering tools to manage the day-to-day stress of school and adolescence more broadly. Given that chronic stress has been identified as a major risk factor for the development of adolescent mental health disorders (Roberts et al., 2009), empowering young people with tools to manage the multiple stressors of this developmental period is an important objective of any health intervention. Difficulties with self-regulation are thought to underlie a variety of psychological disorders, with improvements in self-regulation associated with a reduction of symptoms (Menezes et al., 2015). Previous studies have also suggested potential positive impacts on youths’ health choices and behaviours, such as alcohol and drug use (Butzer et al., 2017; Conboy et al., 2013) but this requires more robust investigation and was not discussed by participants in the current study.

Consistent with previous studies, we found that breathing techniques were the most commonly cited self-regulation practices due to their effectiveness and availability (Conboy et al., 2013; Dariotis et al., 2017; Wang and Hagins, 2016), but meditation and physical poses were also used in multiple contexts. This aligns with previous studies in adults demonstrating the effectiveness of breathing techniques for stress management and mood enhancement, potentially through regulation of the autonomic nervous system (Gard et al., 2014; Schmalzl et al., 2015; Tellhed et al., 2019). Yoga has been associated with reductions in biomarkers of stress, for example, reductions in cortisol levels after a 10-week programme (Butzer et al., 2015) and even after a single yoga session (Sullivan et al., 2019). As demonstrated by youth in the current study, yoga techniques including breathing may be important in improving emotion-focused coping through reappraisal of stressors and reduced physiological arousal (Tellhed et al., 2019). Yoga practices that are easily learnt and implemented in everyday life are likely to be important for the longer-term maintenance of psychosocial benefits and embedding of self-regulation skills in young people.

In line with previous research, participants in the current study described how their yoga practice helped them to build confidence and increase positive feelings about themselves (Wang and Hagins, 2016). However, self-acceptance and self-compassion were primarily discussed in the context of contemplative practices, regardless of physical achievements in yoga. Previous studies have found associations between yoga and increased self-esteem (Bhardwaj and Telles, 2017; Case-Smith et al., 2010) suggesting the value of yoga in supporting the development of a positive self-concept given this plays an important protective role for mental health (Henriksen et al., 2017).

Interestingly, initial barriers to yoga uptake amongst young people primarily related to youth’s self-consciousness and concerns about fitting in. This was magnified by media portrayals of yoga, which has been criticised elsewhere for its ‘exclusionary’ depictions of the ‘yoga body’ creating barriers to uptake (Webb et al., 2020). However, this was contrasted with participants’ actual experiences of yoga which was perceived as inclusive, irrespective of ability, and seen to provide a non-judgemental and supportive environment. In contrast to previous studies (e.g. Conboy et al., 2013), males in our study perceived peers’ engagement with yoga as central to their decisions to take up and maintain their yoga practice – both to avoid feeling ‘gender-swamped’ and to develop their friendships and social networks. Indeed, all participants highly valued the social benefits of yoga, including making and deepening friendships, building social skills and deriving pleasure from sharing yoga with family and friends, as noted elsewhere (Butzer et al., 2017; Conboy et al., 2013; Dariotis et al., 2016; Wang and Hagins, 2016). Consistent with findings in the adult literature, the teacher was perceived to play a key role in facilitating an inclusive, safe space that enabled participants to engage deeply in the yoga practice (Cheshire et al., 2022; Uebelacker et al., 2017).

### Limitations and implications for future research and practice

The current study provided an in-depth perspective of youth’s perceptions and experiences of practising yoga, with rich data saturation. However, there are several limitations to consider. Given that participants were self-selecting, it is likely that they held more positive views about yoga. However, the sample included participants who were no longer practising yoga which provided insights into barriers to ongoing practice. Additionally, the findings were broadly consistent with studies conducted in different settings and contexts suggesting the robustness of the results. A key limitation was lack of diversity in the sample in terms of ethnicity and gender. Whilst this may reflect broader patterns in yoga usage in both youth and adults (Cartwright et al., 2020; Khalsa and Butzer, 2016), future research should consider ways to engage a broader range of young people. In line with previous research on engaging ethnic minority and marginalised groups, our research suggests the importance of emphasising the biopsychosocial benefits of yoga and inclusivity in terms of gender, ethnicity and physical ability in order to counteract negative perceptions of yoga in young people (Spadola et al., 2020; Webb et al., 2020). Further research into cultural influences on perceptions and experiences of yoga amongst youth are also warranted.

Our research has further implications for professionals and educators working with young people. To engage youth in yoga programmes, it is important to highlight benefits that they value – tools to help manage their stress, difficult emotions and mental health; an opportunity to build confidence and social skills in a fun, inclusive and supportive environment. Sharing the stories of young people themselves is a powerful mechanism to do this. A skilled and responsive yoga instructor is essential for youth engagement, enjoyment and trust-formation to facilitate personal and social development. Promotional materials should provide realistic and culturally relevant representations of youth to counteract stereotypes about who practices yoga. For males, a focus on strength and athletic performance may encourage initial engagement, with examples of male role models who practise yoga, such as professional sporting figures, to increase acceptability.

## Conclusions

The present study contributes to a growing body of international research suggesting the effectiveness and acceptability of yoga as a biopsychosocial intervention to support young people’s wellbeing. Importantly it offers new insights into the relational nature of yoga, with practice associated with deepened inter- and intra-personal skills and relationships amongst youth. Within community and school settings, social factors were important in initiating and maintaining yoga practice for both genders. However, lack of male representation underpinned by media portrayals of yoga presented a particular barrier in males. By providing the first in-depth exploration of young people’s perceptions, barriers and experiences of yoga within a UK context, the study contributes to broader discussions around ways to promote adolescent wellbeing in the face of growing demand for mental health support in the UK. An avenue for future research is therefore to evaluate the acceptability and effectiveness of school-based yoga programmes in the UK, in order to increase access and consider optimal dosage. Whilst yoga is a multicomponent intervention, the notable effects, ease and application of yoga breathing techniques suggests an additional area for future research, given that they might be easily integrated into an educational setting with suitable training.

## Research Data

sj-docx-1-hpq-10.1177_13591053221146840 – for ‘It stretches your body but makes you feel good too’: A qualitative study exploring young people’s perceptions and experiences of yoga Click here for additional data file. sj-docx-1-hpq-10.1177_13591053221146840 for ‘It stretches your body but makes you feel good too’: A qualitative study exploring young people’s perceptions and experiences of yoga by Tina Cartwright and Tatjana Doronda in Journal of Health Psychology

sj-docx-2-hpq-10.1177_13591053221146840 – Supplemental material for ‘It stretches your body but makes you feel good too’: A qualitative study exploring young people’s perceptions and experiences of yoga Click here for additional data file. Supplemental material, sj-docx-2-hpq-10.1177_13591053221146840 for ‘It stretches your body but makes you feel good too’: A qualitative study exploring young people’s perceptions and experiences of yoga by Tina Cartwright and Tatjana Doronda in Journal of Health Psychology
