# Supplementary material for: ‘It stretches your body but makes you feel good too’: A qualitative study exploring young people’s perceptions and experiences of yoga
Source: J Health Psychol. 2023 Jan 12;28(9):789–803. doi: 10.1177/13591053221146840 (PMC10387721; doi:10.1177/13591053221146840)
Supplement: sj-docx-1-hpq-10.1177_13591053221146840 – for ‘It stretches your body but makes you feel good too’: A qualitative study exploring young people’s perceptions and experiences of yoga [file sj-docx-1-hpq-10.1177_13591053221146840.docx]

**Supplementary file**

**Themes and Subthemes: Illustrative Quotes**

| *Themes* | *Subthemes* | *Indicative Quotes* |
| --- | --- | --- |
| **1. Yoga as a “Mind-Body Thing”** |  | *“I found it quite nice in terms of a body thing, cause obviously you are stretching and you are working your muscles in terms of that you feel quite good. And especially if you have a lot of worries or you are stressed out, it makes you feel nice in that way when you clear your head of everything at the end.”* FG 3  *“I think yoga is like… physically calming your body, but also mentally. Like... when you feel physically calm, you are mentally calm.”* FG 8  *“I think it’s exercise but it helps you focus your mind stuff like that, and calm you down… but it’s also like for your body, and for your mind.”* FG 2  *“Yes, to me personally it is more psychological rather than physical exertion. I know it can be physically challenging but I focus more on a psychological aspect of it.”* FG 5  *“…yoga it’s challenging and it’s good for your physical health, but it’s also like, good for your mental health and it, like, relaxes you… FG 6*  *“So, yoga I think is… for me… I think it’s the form of everything…. I think that you can use it as a form of exercise, but for me it’s been like calming me down and like, relaxing me, but doing it in the way that doesn’t mean you just sit on the phone and watch Netflix.”* FG 10 |
|  | 1.1 Finding the "sweet spot": mindful awareness | *“We got taught how in meditation you are trying to find that space between going to sleep and being awake, you find like a ‘sweet spot’ in between.” FG 3*  *“…you sort of go from this massive busy day, with so much going on… to being clear and mindful.”*  *Researcher: “What do you mean by being ‘mindful’?”*  *“Pretty much being aware of your surroundings. The given word sort of contradicts the actual meaning. It’s like mind full of stuff, but actually it’s just being focused on what is around you from all the senses.” FG 1*  *“[yoga brings] to almost that neutral state of body and mind, cause you are kind of between tired and alert, in my terms… Your body isn’t…. exhausted but you are not energized either. So, it sort of centers you almost – which I found really nice. It’s quite a consistent thing and it generally bring you back to a similar point of calm.” FG3*  *“Well, I always feel a lot more awake and like, aware… It’s almost like, you kind of relaxed yourself but also, it’s not like a sleepy relaxed, it’s like, refreshed feeling.” FG6*  *“For me yoga is more about… it’s like another kind of form of escapism. Yeah... it does kind of take you into another place where you do… it just like kind of roots you in a moment.” FG 5*  *“I feel like, it’s very like, spiritual and It’s connecting you with your spirit… so, in opposition to rugby and stuff which is a high intensity, this is like, it brings you to the… it’s grounding… and it’s also like, connecting with yourself.”*  FG 10 |
|  | 1.2 Enhancing physical performance | *“I like it, cause I am a dancer, so I like doing yoga to calm me down before like a performance or like, in my warm-up at home I do stretches and it just helps.”* FG5  *“And also, when I do like, running or something, it’s made a big difference, because it doesn’t hurt when I run, it makes it a lot more fluid almost, because I’m not tight or anything.”* FG 6  *“Cause I do my dancing it helps get more flexible and also like, my shoulders get quite tight when I do swimming and it just gets me a lot more flexible, so I can do a lot more of it.” FG 7*  *“I had problem with my spine, so it curves… so, I came to yoga so it stops curving as quickly… so when I go to see the doctor, he is telling me that yoga is helping and it’s good because I enjoy doing yoga as well.”* FG 7  *“…let’s say this week I was on DofE (Duke of Edinburgh) expedition which is like, quite physically enduring, like walking for like, 4 days. At night when we were all like tired and everything aches, we used yoga a lot and I showed my friends also different poses that I do in the class and they definitely benefit from it as well, like me, practicing outside the class.”* FG 8  *“I mean, I don’t really like, use it a lot. If I’m like, during PE if we are about to do like a …like race or something… I’ll like breathe deeply, but for more like, physical reasons to relax my body, cause if it’s a long run, it concerns your breathing as well.” FG 9* |
| **2. Yoga for Empowerment and Self-regulation** |  | *“Instead of having a closed mind set, yoga can help you develop more of an open one and see the better sides of things. Like, it can make harder situations easier, like things that you struggle with every day it would help you make that a lot easier and give you sort of push.” FG 2*  *“…So, having had that being rooted at some point, I found it easier to kind of look at it: “no, this situation is ok”, cause I know I can just walk away for a minute, do something else.“ FG 5*  *“I think, to me, like, personally yoga has really helped. I go to therapy for situations and I think yoga for me has been more empowering like, I feel like, I’ve been in more control of what my mind is thinking….it’s very important for young people to feel like, no matter the situation, like they are in control of what they can do.” FG 8* |
|  | 2.1 “I feel a lot better about myself” | *“It makes you feel like… For me personally, I feel more open…more comfortable with myself, so like… I feel a lot better about myself.” FG 2*  *“Before I came to yoga, I used to get quite frustrated a lot, like at home and after school when something happened…. But when I came to yoga, I started to feel better about myself and it made me a little bit more confident. So, now I don’t get as upset and if I do, I just do some breathing and do some poses…” FG 7*  *It makes me feel calm and it makes you feel like it doesn’t really matter what anybody else thinks of you, because you are doing it as best as you can. FG 7*  *“Like sometimes when you’ve had a really stressful day at school and someone made you feel upset. Then you are feeling a bit down with yourself… Or maybe you’ve said something that you think you shouldn’t have said or something. Like, it would make you feel better about yourself cause you are being like, hard on yourself.” FG 4*  “*You kind of, instead of thinking - oh, I need to push myself harder! It’s more like - oh, I just need to be thankful for what I’ve done! This kind of makes you feel proud almost because you’ve done something for yourself.”* FG 6  *“…. just feeling physically, like a bit fitter with that regular practice, it’s just quite nice, cause you’re thinking “oh, yeah, I’m doing quite well”. I guess it just helps building that self-confidence.” FG 5* |
|  | 2.2 Stress and emotional regulation | *“Yeah, I do enjoy practicing yoga, cause it makes me feel less stressed, like sometimes I get quite stressed out about things, erghm, and it just makes me feel more relaxed…. especially in the meditation as well. And, yeah.”* FG4  *“I agree with others, relaxation is probably my favorite part about yoga… cause if you have something worrying you in your mind it just sort of lets it all go away. And you can just relax and have some time to yourself to think things through.” FG2*  *“It gets you ready. Although at the end it calms you down, it still keeps you quite active. So, you feel like you are ready for the day, I think.” FG4*  *“I would definitely recommend it as well. Quite similar again, but just for that thing where it takes you completely out of your mind space. Especially with those times when you are going through all of it, like with A-levels - it’s stressful! GCSE – it’s ridiculously stressful!! Like most of the school process can be quite a stressful time. Cause most of the young people are still in education. It’s such a good thing to be doing just to completely kind of have a mechanism to relax and calm yourself for the entire thing.” FG 5*  *“because some of the stuff that we do is quite challenging and it makes you, like, think about things that you’re doing instead of thinking about other things…” FG 8*  *“It depends on the situation after yoga, what’s going on… so, it depends if I keep that relaxed state. So, it can sort of be easily jogged, but it definitely lowers my stress levels. And it’s quite nice after a day of when you are exhausted, it helps to calm the mind.” FG 1*  *“Personally, I feel like it can go quite fast [the effects]. I will do it for a certain amount of time, whether short or long and I will come out of it and something will happen which will just sort of you know, make me feel a bit different and I won’t feel that calm, you know…. And doing yoga after that can really help your mind and is very good for mental health.” FG 1* |
|  | 2.3 Toolkit for self-regulation | *“Yeah, I feel like breathing is one of the yoga’s main thing, and meditation. You can use them at school or at work, go to the toilets, and just sort of breathe or meditate and you know, even just do it at class, wherever you are.” FG 1*  *“I feel like for the people who are listening to the sound, it teaches them awareness to try and block out all the other sounds and listen for that certain little sound. It sort of teaches you to focus your mind on that little sound.” FG 1*  *“Yes, also breathing through your tummy is also quite good. They also told us…about making your in-breath longer than your out-breath gives you more energy and making your outbreath longer than in-breath relaxes you. I’ve retained that quite a bit even in non-yoga related stuff, like sports, like…sometimes if I’m out for a run if keep my inbreath longer and that’s giving me more energy. It’s helpful in other ways, it’s quite educational as well.” FG3*  *“Well, before yoga when I was feeling anxious, I didn’t really know how to deal with it, but after it I learnt how to breathe. Like, I already knew how to breathe, but like…in my nose and out of with my mouth to help control those feelings. Yeah.” FG 4*  *“Our yoga teacher says if we are feeling stressed and we just want to like calm down we can go somewhere quiet and you can just practice that breathing. I think doing that can just... I think as well when you are stressed you can do stuff you probably don’t mean. So, then you can just calm down a little bit and then you will be more yourself, calm.” FG 4*  *“So, now I don’t get as upset and if I do, I just do some breathing and do some poses…” FG 7*  *“I think that yoga helps before exams, because of all the build-up of tension… you sometimes find yourself getting like, overwhelmed… so, breathing techniques can help in a lots of situations… just to calm you down and make sure that you don’t get overstressed, so that you don’t do as well as you could in the exam….” FG 10*  *“I quite like the breathing. I find that when you’ve got like, really like… busy mind, when you’re breathing you only like, think of you breathing. I think it’s a really good distraction off the things that are like making me feel, like overwhelmed or… yeah. And I think doing that at the beginning and at the end of yoga… like, helps you get ready for the actual yoga.”*  *“Yeah, I feel like emotions when they are running hype… they are also helpful, to like, just the breathing techniques again. It just brings you… and calms you down… If you are angry, it’s there… I think it does help.”*  *“So, often when I am in pain, like really bad pain, I’ll focus on breathing techniques, even though it’s like, not the first thing that’s in my mind. But when you take a step back, that’s one thing I do and my younger sister suffers from like, really bad groin pain at night, so she can’t sleep. And one thing we’ve done recently is... we’ve started doing like, breathing techniques that I’ve learnt from yoga and even like, different yoga poses and it’s made a massive impact that we didn’t think would happen.” FG 10* |
| **3. The Social Context of Yoga** |  |  |
|  | 3.1 Misconceptions about yoga and barriers to practice | *“My friends did go there, but then slowly they dropped out. I was the only guy. Maybe if they hadn’t dropped out, I would be still doing it today”. FG 1*  *“Well, lot of people care a lot about the social aspect of school… like when people changing in the changing room people would be sort of, you know “haha, you guys are going to yoga, how cool” (skeptically).*  FG 3  *“Yeah, sometimes my friends at school they are just… they are saying that yoga is like, really easy, it’s like standing still and…. Just like doing really easy things… and they think it’s boring. And it isn’t, they just assume…”*  *“My friends today, I was speaking to them and I was like “**I can’t play with you because I’ve got yoga”, and they said “yoga – that’s for old people!” And I said to her… And she wants to be a professional dancer and she is not flexible…’if you want to be a professional dancer, you want to be flexible and it [yoga] would just help you’” FG 7*  *“… none of my other friends know that I come here, but if they did they’d think it would be kind of weird – they’d be like ‘oh, yoga, like, that’s a bit weird… why don’t you go to like, football practice or something like that instead’.” FG9*  *“Someone might feel like they might not be physically and mentally flexible to do it. They might be like: “Oh, yeah but if I go, I might not be able to do this and do that”. And this keeps this sort of mindset and they can’t open themselves to do it and it might be a set-back.” FG2*  *“Yeah, I agree with X as well, as some people might think they won’t be good enough to do it or that people would judge them, so that’s one barrier.”*  *“…Cause there is that barrier in which there is that fear of the new, fear of the unknown. That kind of gets in a way and there is like “Argh, I would do it, but then I don’t want to be in a class surrounded by other people and then they all would be doing it better than me, and then if I mess up on this they would all laugh at me and stuff like that.”* FG 5  *“I was really scared of being judged when I went, cause I’m not like the most flexible person, I can’t do everything… So, I was really scared of being judged. But it’s better now when you go every week and you get used to it.”* FG 8  *“I think, one thing… at the beginning when I started yoga, one thing that stopped me was worrying about who would turn up to yoga… like, I’m very happy with like, who goes to yoga now…. but at the beginning I was like a bit like nervous, I didn’t really know… like, who’s gonna be there. And if that wouldn’t have turned out to be like, a positive thing, I wouldn’t enjoy going to yoga, I don’t think. Knowing that I’ve got people that don’t judge, or like… don’t kind of… we all kind of focus on our own thing in yoga. And that is really helpful… like I’m not worried about anything to do with people side of it… FG 8* |
|  | 3.2 Inclusivity: “*It’s a big community thing”* | *“The big thing about yoga it’s not competitive. It’s more of a… You kind of help each other in way. It’s a big community thing. You invite everyone in, there is no exclusion of anyone. That’s how we experience it.*  *Yeah.*  *It’s good for making friends in that respect.*  *I agree, it is non-competitive, it’s almost cooperative!”*  *“We had two teachers one of which wasn’t very structured and you just talk whilst doing it and just you would help each other that way. We did actually do stretches with each other and worked in groups on things. Emm… yeah, it’s more cooperative than competitive, it sort of… it’s quite difficult but you don’t generally get physically exhausted. You aren’t panting your breath or anything like that, so in that respect it’s different from sports.” FG 3*  *“I would recommend it, it was very useful! Something that did help a lot - the first time I went with a friend, so if you can find a buddy who is willing to go as well, that helps a lot.” FG 4*  *“Yoga, it’s very like, Zen… so, it’s very calming and you don’t really think about… like, when running you may think about aggression or rugby…. But I think with yoga, I think you just… you don’t think about aggression, you think like, about another side of you… and I think it brings out a lot of good in people.”* FG 10  *“ I was thinking it’s like, it’s quite a social event as well, cause you chat to your friends and it’s quite nice to meet up while you are also exercising and relaxing.”* FG 10  *“And also, our teacher, she knows her job and how to do it well. She is like shared stories about herself, and that helps us open us open out as well and like, we can trust her more. And it makes us enjoy it more and it makes it more real… rather than just some random person who walks in: ‘ok eyes closed, lie down…’ FG 9*  *“I like the partner poses, cause it can push you to your limits and it can help you like, become stronger… and go even further because you can partner up with someone who has a higher limit and they can help you get higher.” FG6*  *“Yeah, it can help you with like, with your social life, cause you have to talk to other person and trust that they know what they are doing.”*  *“Some people do it because they lie about it… they say that they don’t wanna go… but actually it’s because like, something’s happened or maybe none or the friends are going and they just don’t wanna go. But if you go, it can calm you down and you can make new friends*.” *FG 7* |
