# Supplementary material for: ‘It stretches your body but makes you feel good too’: A qualitative study exploring young people’s perceptions and experiences of yoga
Source: J Health Psychol. 2023 Jan 12;28(9):789–803. doi: 10.1177/13591053221146840 (PMC10387721; doi:10.1177/13591053221146840)
Supplement: sj-docx-2-hpq-10.1177_13591053221146840 – Supplemental material for ‘It stretches your body but makes you feel good too’: A qualitative study exploring young people’s perceptions and experiences of yoga [file sj-docx-2-hpq-10.1177_13591053221146840.docx]

**Supplementary material**

**Table 1: Participants’ Characteristics**

| **Pseudonym** | **Age** | **Gender** | **Practice length (months/years)** | **Practice frequency** |
| --- | --- | --- | --- | --- |
| Lea | 15 | F | 3 years | Once a week |
| Olivia | 15 | F | 2 years | 2-3 times a week |
| Sam | 13 | F | 18 months | Once a week |
| Willow | 14 | F | 2 years | Once a week |
| Maxeine | 14 | F | 2 years | Once a week |
| Michelle | 10 | F | 6 months | Twice a week |
| Carla | 13 | F | 3 months | Once a week |
| Olivia | 17 | F | 10 months | Once a week |
| Kate | 15 | F | 2 months | Once a week |
| Grace | 14 | F | 4 months | Once a week |
| Alice | 13 | F | 4 months | Once a week |
| Tim | 18 | M | 6 months | Once a week |
| John | 10 | M | 2 months | Twice a week |
| Claire | 13 | F | 10 months | Once a week |
| Adam | 12 | M | 1 year | Once a week |
| Bob | 10 | M | 10 months | Once a week |
| Quentin | 15 | M | 3 months | Once a week |
| Jazmin | 10 | F | 9 months | Twice a week |
| Harriet | 11 | F | 15 months | Once a week |
| Tillyboo | 10 | F | 5 months | Once a week |
| Vector | 14 | M | 1 year | Once a week. Not currently |
| Bobbie | 10 | F | 1 year | Once a week |
| Tia | 10 | F | 3 months | 3 times a week |
| Alan | 15 | M | 1 year | Once a week. Not currently |
| James | 15 | M | 1 year | Once a week. Not currently |
| Evie | 11 | F | 6 years | Daily |
| Rebecca | 13 | F | 3 months | Once a week |
| Mary | 12 | F | 3 months | Every week |
| Eloise | 13 | F | 5 months | Once a week |
| Millie | 11 | F | 4 months | Once a week |
| Lola | 12 | F | 2 months | Once a week |
| Sophie | 13 | F | 2 months | 1-2 times a week |
| Gertrude | 11 | F | 4 months | Once a week |
| Jamie | 15 | F | 3 months | Once a week |
| Charlie | 12 | F | 18 months | Once a week |

Focus Group Schedule

- Do you enjoy practicing yoga? Why?
- What does yoga mean to you personally? What is your definition of yoga?
- How does yoga make you feel? What have you found are the main effects of yoga? (physical/emotional/psychological)?
- Has attending yoga classes changed you in any way? Body/mind/attitudes?
- What aspects of a yoga class do you enjoy most (breathing exercises, asana, meditation, relaxation, social interaction, specific poses) and why?
- What is your least favorite part of the practice?
- Do you use any yoga techniques in daily life, outside of the class? Do you use particular techniques? When do you apply them?
- What do you think could be the barriers to yoga practice? (what do others think about your yoga practice)?
- Would you recommend yoga to other young people? Why?
